# Polyethylene Micro/Nanoplastics Exposure Induces Epithelial–Mesenchymal Transition in Human Bronchial and Alveolar Epithelial Cells

**DOI:** 10.3390/ijms251810168

**Published:** 2024-09-22

**Authors:** Alice Traversa, Emanuela Mari, Paola Pontecorvi, Giulia Gerini, Enrico Romano, Francesca Megiorni, Amedeo Amedei, Cinzia Marchese, Danilo Ranieri, Simona Ceccarelli

**Affiliations:** 1 Department of Life Sciences, Health and Health Professions, Link Campus University, 00165 Rome, Italy; a.traversa@unilink.it (A.T.); e.mari@unilink.it (E.M.); 2 Department of Experimental Medicine, Sapienza University of Rome, 00161 Rome, Italy; paola.pontecorvi@uniroma1.it (P.P.); giulia.gerini@uniroma1.it (G.G.); francesca.megiorni@uniroma1.it (F.M.); cinzia.marchese@uniroma1.it (C.M.); simona.ceccarelli@uniroma1.it (S.C.); 3 Department of Sense Organs, Sapienza University of Rome, 00161 Rome, Italy; enrico.romano@uniroma1.it; 4 Department of Experimental and Clinical Medicine, University of Florence, 50121 Florence, Italy; amedeo.amedei@unifi.it

**Keywords:** polyethylene, EMT, bronchial, alveolar, MNPs

## Abstract

Micro/nanoplastics (MNPs), which are widely spread in the environment, have gained attention because of their ability to enter the human body mainly through ingestion, inhalation, and skin contact, thus representing a serious health threat. Several studies have reported the presence of MNPs in lung tissue and the potential role of MNP inhalation in triggering lung fibrosis and tumorigenesis. However, there is a paucity of knowledge regarding the cellular response to MNPs composed of polyethylene (PE), one of the most common plastic pollutants in the biosphere. In this study, we investigated the effects of low/high concentrations of PE MNPs on respiratory epithelial cell viability and migration/invasion abilities, using MTT, scratch, and transwell assays. Morphological and molecular changes were assessed via immunofluorescence, Western blot, and qRT-PCR. We demonstrated that acute exposure to PE MNPs does not induce cellular toxicity. Instead, cells displayed visible morphological changes also involving actin cytoskeleton reorganization. Our data underlined the role of epithelial–mesenchymal transition (EMT) in triggering this process. Moreover, a remarkable increase in migration potential was noticed, in absence of a significant alteration of the cell’s invasive capacity. The present study highlights the potential impact of PE MNPs inhalation on the human respiratory epithelium, suggesting a possible role in carcinogenesis.

## 1. Introduction

Despite the effort to develop and employ more ecological and sustainable materials, plastics are still broadly used in many fields of the industry for producing and packaging a plethora of human manufactures. Indeed, plastic possesses some characteristics, such as durability and flexibility, which make it suitable for various applications. Moreover, the plastic manufacturing process is generally efficient and less expensive than other materials like metal or glass. As for product packaging, plastic represents a good choice due to its ability to provide a barrier against contaminants and its lightweight nature that can reduce transportation costs [1].

However, the massive use of plastic in everyday appliances is raising concerns and interest in the potential effects of plastic contamination on both environmental and human health. Such concerns have been especially evident during the COVID-19 pandemic where a huge accumulation of disposable personal protective equipment has been released into the environment [2].

Plastics dispersed in terrestrial and aquatic environments are exposed to biological, chemical, and physical conditions that cause them to be broken up into microplastics and nanoplastics, defined as particles with a diameter of less than 5 mm and 1 μm, respectively. As a consequence, micro/nanoplastics (MNPs) are now responsible for a global environmental contamination, which has significant repercussions on the health of ecosystems and animal species [3], including humans [4]. In fact, MNPs can enter the food chain and reach humans through ingestion, inhalation, and skin contact, leading to their accumulation in various tissues and organs, such as the blood, lungs, and placenta [5]. MNPs have been reported to have genotoxic and cytotoxic effects, inducing oxidative stress, DNA damage, inflammation, and immune responses, ultimately causing carcinogenesis in humans [6]. Moreover, MNPs have been detected in atherosclerotic plaques, suggesting a possible role in cardiovascular diseases [7]. In addition, because of their high surface area, MNPs can harbor or absorb many different types of chemicals and pathogens from the environment.

To date, limited studies addressing MNPs’ effects on humans are available, as most of them are focused on high-density plastics, such as polystyrene (PS) and polyvinyl chloride (PVC). Besides the chemical composition of MNPs, other challenging issues in this research field are the lack of standardized analytical methods and the variability of key factors, such as MNPs concentration, diameter, exposure route, and duration [8]. Therefore, further studies are mandatory to understand the real impact of MNPs on human health.

In particular, inhalation of MNPs is a privileged route for systemic uptake [9]. Depending on their size and shape, inhaled MNPs can accumulate in the lungs and be internalized by alveolar epithelial cells [10,11], thus representing a potential risk factor for respiratory diseases. Studies using PS MNPs have demonstrated their accumulation, inflammatory effects, cytotoxicity, and reduced cell viability in human lung and alveolar epithelial cells [12,13]. Also, polyethylene (PE) MNPs have previously shown the ability to accumulate in human organs, including the lungs [14]. However, besides the PE MNPs abundance in the atmosphere, the specific effects of PE MNP inhalation on human lung tissues still need to be deeply investigated. In this work, we aimed to assess the effects of acute exposure to PE MNPs in human alveolar and bronchial epithelial cells, focusing on epithelial–mesenchymal transition (EMT), and on the migratory and invasion ability of the two cell lines, in an attempt to shed light on the potential role of MNPs in lung carcinogenesis.

## 2. Results

### 2.1. Acute Treatment with PE MNPs Alter Cell Morphology but Does Not Impact Cell Proliferation and Viability in Human Bronchial and Alveolar Epithelial Cells

**Figure 1:**
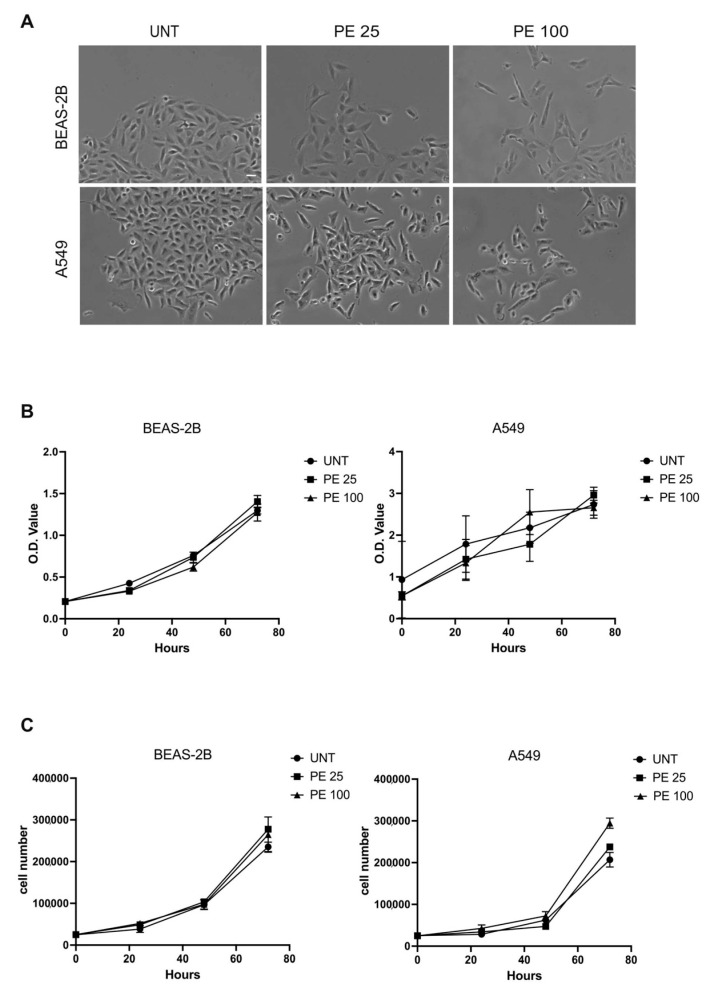
Evaluation of acute PE MNPs exposure on morphological characteristics cellular viability and proliferation in human bronchial epithelial cells (BEAS-2B) and human alveolar epithelial cells (A549). (**A**) Representative phase contrast images of BEAS-2B and A549 cells treated for 24 h with 0.01% Tween20 (UNT) or PE MNPs at 25 μg/mL (PE 25) and 100 μg/mL (PE 100). Bar: 20 μm. (**B**) The effect of PE MNPs at (24, 48, and 72 h) on BEAS-2B and A549 cell viability as determined via MTT assay and expressed in O.D. value. (**C**) The effect of PE MNPs at 24, 48 and 72 h on BEAS-2B and A549 cell proliferation, as determined via viable cell counts. (**B**,**C**) Mean value ± standard deviation (SD) was obtained from three independent experiments, each performed in triplicate.

Starting from previous studies assessing PE MNPs effects on vaginal epithelial cells [15], we investigated their impact on human bronchial epithelial cells (BEAS-2B) and human alveolar epithelial cells (A549). After 24 h of treatment with 25 μg/mL and 100 μg/mL of PE MNPs, we observed morphological changes in cells treated with both doses, with respect to the untreated ones. Phase contrast images showed that while untreated cells appeared closely packed and organized in compact colonies of polygonal cells, treated cells appeared spindle-shaped and detached from the neighboring ones (Figure 1A). After 24 h of treatment, we did not highlight significant alterations of cell cycle phases in treated cells with respect to controls.

Then, we evaluated the effect of PE MNPs on the same cell lines in terms of toxicity, and therefore the impact on cell viability and proliferation at longer times. We exposed the cell lines for 24, 48, and 72 h to PE MNPs treatment. The MTT test did not show significant differences in the growth curve of the two cell lines between cells exposed to plastics and untreated controls (Figure 1B). Additionally, direct cell counts displayed no significant difference in proliferation between treated and untreated cells at 24, 48, and 72 h for both cell lines (Figure 1C). At 72 h, a slightly increased proliferation compared to the untreated was visible only for A549 cells treated with PE 100, and not for BEAS-2B cells (Figure 1C).

**Figure 2:**
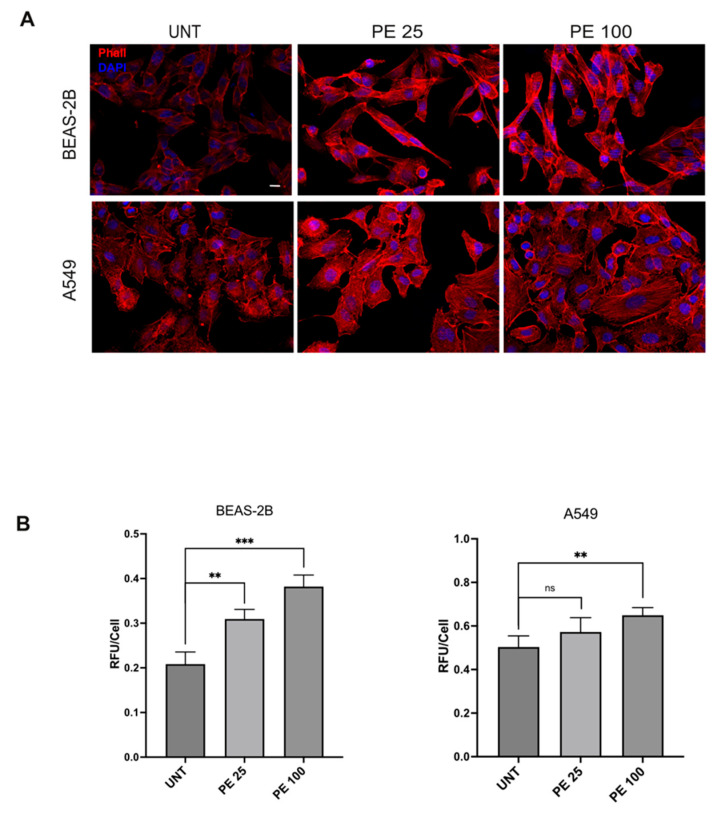
Immunofluorescence analysis of the effects of acute PE MNPs exposure on actin cytoskeleton architecture in BEAS-2B and A549 cells. (**A**) Representative IF acquisitions (N = 3) showing actin cytoskeleton of BEAS-2B and A549 cells treated with PE MNPs (PE 25 and PE 100) or untreated (UNT). F-Actin is stained in red with TRITC-Phalloidin and nuclei are stained in blue with DAPI. Bar: 10 μm. (**B**) Quantitative immunofluorescence analysis of TRITC-Phalloidin staining, expressed as the mean ± SD relative fluorescence unit per cell (RFU/cell). ns, not statistically significant; **, *p* < 0.01; ***, *p* < 0.001.

In order to further unravel the effects of acute 24 h PE MNPs treatment on bronchial and alveolar epithelial cell morphology, the architecture of the actin cytoskeleton was analyzed via fluorescence analysis using phalloidin-TRITC. The results showed that, in agreement with the compact appearance of colonies observed via phase contrast microscopy, untreated cultures displayed cobblestone-shaped and tightly packed cell organization in which the actin cytoskeleton appeared distributed in peripheral cortical filaments (Figure 2A). In contrast, in PE MNPs-treated cell lines, despite the presence of cortical bundles, several peripheral cells appeared with an elongated shape, away from neighboring cells and with a strong actin cytoskeleton reorganization, in both cell lines and PE MNPs concentrations. Especially in A549 cells, as expected, given the transformed nature of the cell line, the actin cytoskeleton formed thick bundles of stress fibers in some treated cells (Figure 2A). Thus, differently from untreated cells, acute PE MNPs exposure appeared to affect both the cell lines’ morphology and their ability to grow in packed and compact colonies. Quantitative immunofluorescence analysis in BEAS-2B cells showed that phalloidin signal staining intensity, expressed as mean relative fluorescence units per cell, was increased after 24 h of treatment with 25 μg/mL PE MNPs, and to a greater extent in the same cells treated with 100 μg/mL PE MNPs. Similarly, the actin staining signal measured in A549 also showed an increasing trend in the 25 μg/mL treatment, although not statistically significant, and a significantly higher value in the same cells exposed to the 100 μg/mL concentration (*p* < 0.01) (Figure 2B).

### 2.2. PE MNPs Exposure Induces Modulation of EMT Markers

**Figure 3:**
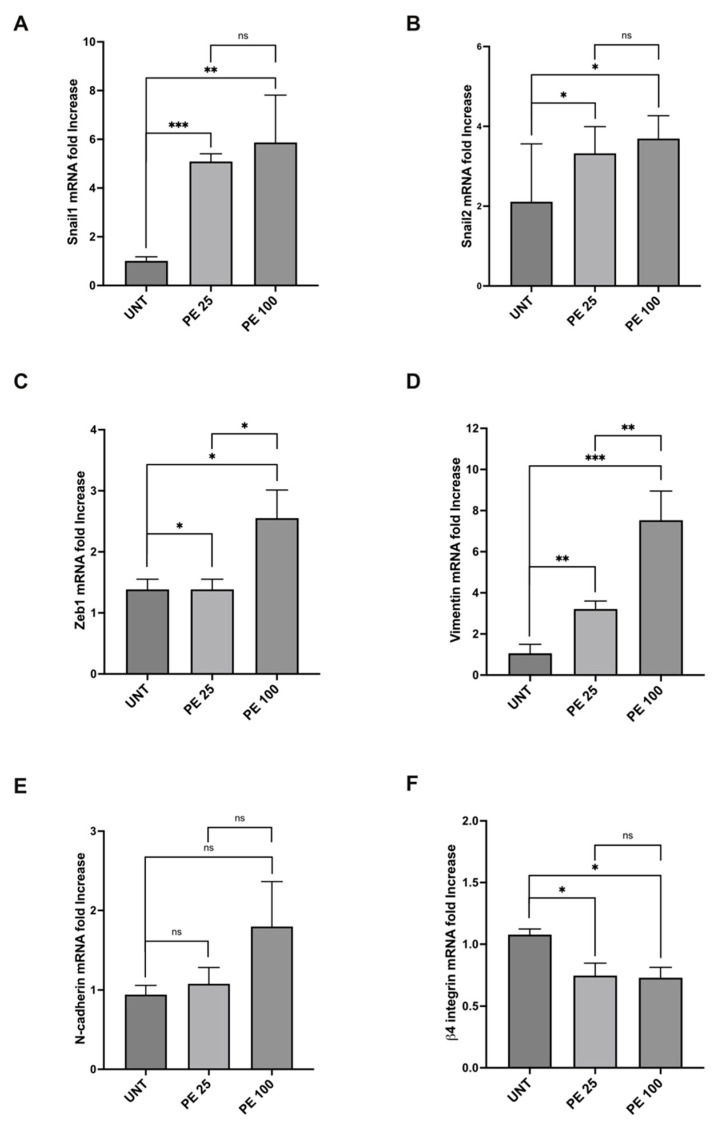
Modulation of mRNA expression levels of EMT markers after acute PE MNPs exposure in BEAS-2B cells. (**A**–**F**) Bar graphs (N = 3) showing gene expression levels of the EMT markers Snail1 (**A**), Snail2 (**B**), Zeb1 (**C**), and of the epithelial/mesenchymal markers vimentin (**D**), N- cadherin (**E**), β4-Integrin (**F**) in BEAS-2B cells, treated for 24 h with PE MNPs (PE 25 and PE 100), compared to untreated cells (UNT). Results are expressed as mean value ± SD. ns, not statistically significant; *, *p* < 0.05; **, *p* < 0.01; ***, *p* < 0.001.

**Figure 4:**
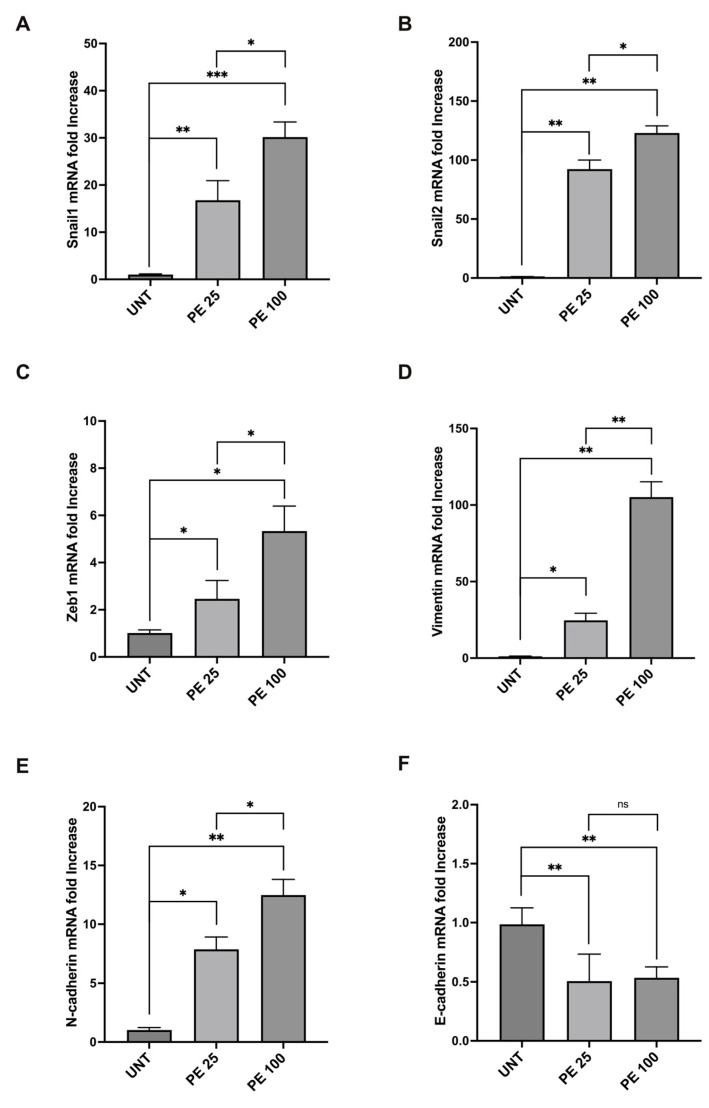
Modulation of mRNA expression levels of EMT markers after acute PE MNPs exposure in A549 cells. (**A**–**F**) Bar graphs (N = 3) showing gene expression levels of the EMT markers Snail1 (**A**), Snail2 (**B**), Zeb1 (**C**), and of the epithelial/mesenchymal markers vimentin (**D**), N-cadherin (**E**), E-cadherin (**F**) in A549 cells, treated for 24 h with PE MNPs at low and high concentrations (PE 25 and PE 100), compared to untreated cells (UNT). Results are expressed as mean value ± SD. ns, not statistically significant; *, *p* < 0.05; **, *p* < 0.01; ***, *p* < 0.001.

The morphological variations induced via PE MNPs treatment in both BEAS-2B and A549 cell lines showed a similarity to those occurring during the epithelial mesenchymal transition (EMT) process, suggesting that the PE MNPs exposure could be able to alter the mRNA expression pattern of epithelial/mesenchymal biomarkers. To assess if PE MNPs exposure was able to initiate EMT in our cellular models, we investigated the mRNA expression of Snail1, Snail2, and ZEB1, all well recognized master transcription factors that are mainly related to the early steps of the process [16]. Real-time RT-PCR demonstrated that the treatment with PE NMPs at both concentrations tested was accompanied by a significant increase in Snail1, Snail2 and ZEB1 in both BEAS-2B (Figure 3A–C) and A549 cells (Figure 4A–C). In addition, the treatment at 100 μg/mL was capable of inducing gene upregulation more than the treatment at 25 μg/mL for ZEB1 in BEAS-2B (Figure 3C), and for all the transcription factors under examination in A549 cell line (Figure 4A–C), emphasizing a dose-response action. Thus, consistently with the hypothesis of a role for PE MNPs treatment in the switch towards a mesenchymal phenotype, real-time RT-PCR was performed to evaluate vimentin and N-cadherin mRNA expression. Gene expression study showed that the mesenchymal marker vimentin was induced in both BEAS-2B (Figure 3D) and A549 cells (Figure 4D) with a response dependent on the treatment dose. Instead, the expression of the N-cadherin marker was increased only in A549 (Figure 4E) and not at all influenced by the treatment in BEAS-2B (Figure 3E). Finally, in line with our hypothesis of a morphological variation driven by a genetic reprogramming attributable to EMT, we tested the gene expression of two epithelial markers, β4-integrin in BEAS-2B and E-cadherin in A549 cell line, which both showed a significant decrease given by the treatment with PE NMPs (Figure 3F and Figure 4F).

**Figure 5:**
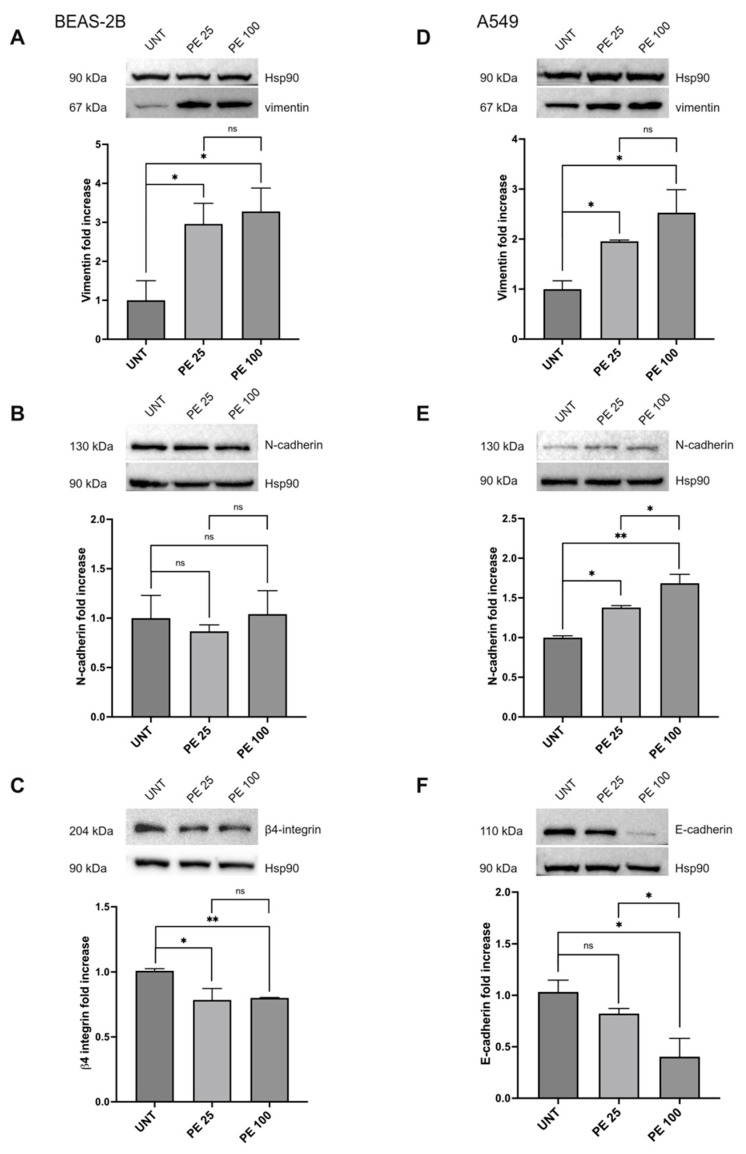
Protein expression analysis of EMT markers after acute PE MNPs exposure in BEAS-2B and A549 cells. (**A**–**C**) Representative Western blot analysis (N = 3) for the EMT markers vimentin (**A**), N-cadherin (**B**), β4-integrin (**C**) in BEAS-2B exposed to PE MNPs (PE 25 and PE 100) for 24 h, compared to untreated cells (UNT). HSP90 was used as loading control. Densitometric analysis was reported as relative expression with respect to untreated cells. Results are expressed as mean value ± SD. ns, not statistically significant; *, *p* < 0.05; **, *p* < 0.01. (**D**–**F**) Representative Western blot analysis for the EMT markers vimentin (**D**), N-cadherin (**E**), E-Cadherin (**F**) in A549 exposed to 25 μg/mL and 100 μg/mL of PE MNPs (PE 25 and PE 100) for 24 h, compared to untreated cells (UNT). HSP90 was used as loading control. Densitometric analysis was reported as relative expression with respect to untreated cells. Results are expressed as mean value ± SD. ns, not statistically significant; *, *p* < 0.05; **, *p* < 0.01.

The modulation of vimentin, N-cadherin, E-cadherin, and β4-integrin expression was further investigated via Western blot analysis (Figure 5). The results clearly showed an increase in the mesenchymal marker vimentin for both cell lines (Figure 5A,D), while regarding N-cadherin, the increase was statistically significant only in A549 cells (Figure 5E), as expected given the gene expression data, while it was not perturbed by both PE MNPs treatments in BEAS-2B (Figure 5B). In regards to the epithelial markers, both the β4-integrin in the BEAS-2B cell line (Figure 5C) and the E-cadherin in the A549 cell line (Figure 5F) were negatively modulated by the treatments. E-cadherin, in particular, also showed a dose-related decrease (Figure 5F).

### 2.3. The Effects of PE MNPs Exposure on Cell Morphology and EMT Induction Can Be Reverted after PE MNPs Removal 

**Figure 6:**
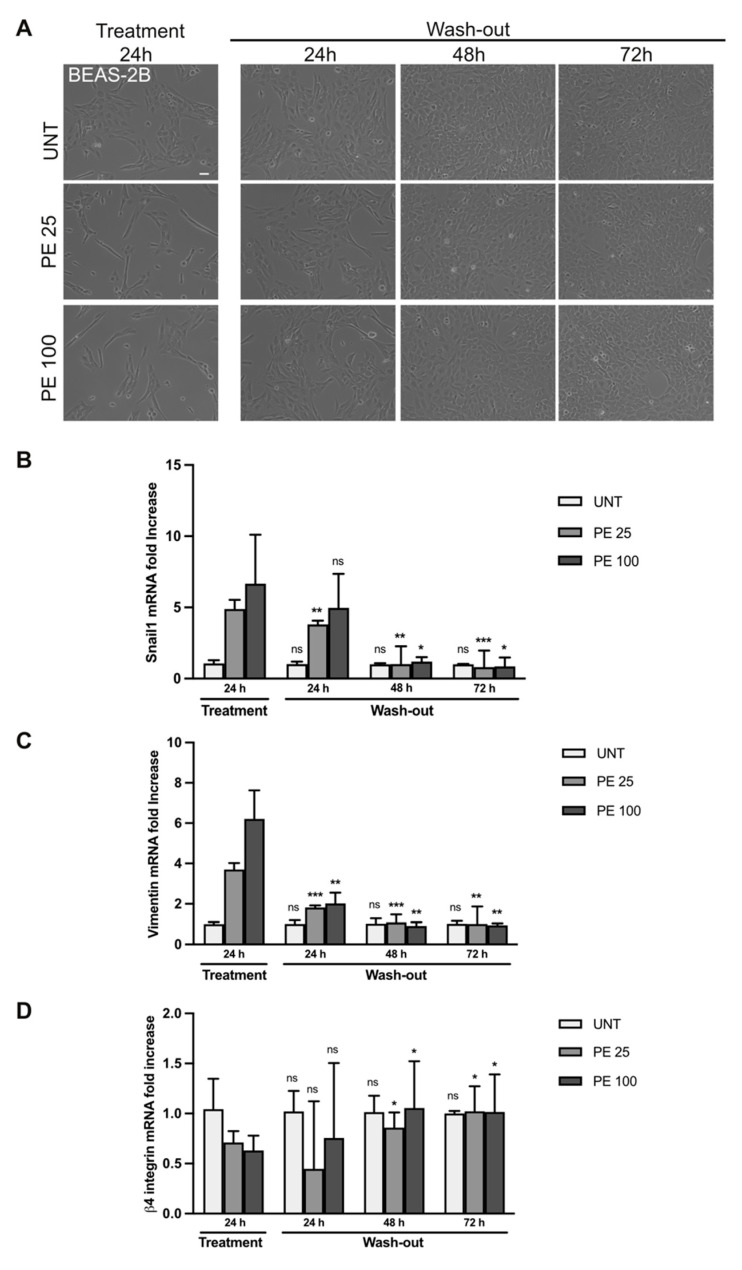
Rescue of PE MNPs-induced EMT phenotype in BEAS-2B cells. (**A**) Representative phase contrast images of BEAS-2B cells untreated (UNT) or treated with PE-MNPs (PE 25 and PE 100) for 24 h and then washed to remove PE-MNPs and cultured in standard medium for 24, 48, and 72 h. Bar: 20 μm. (**B**–**D**) Bar graphs (N = 3) showing gene expression levels of the EMT marker Snail1 (**B**), and of the epithelial/mesenchymal markers vimentin (**C**) and β4-Integrin (**D**) in untreated BEAS-2B cells or treated with PE-MNPs for 24 h and after 24, 48, or 72 h of wash-out. Results are expressed as mean value ± SD. ns, not statistically significant; *, *p* < 0.05; **, *p* < 0.01; ***, *p* < 0.001 vs. treatment 24 h.

**Figure 7:**
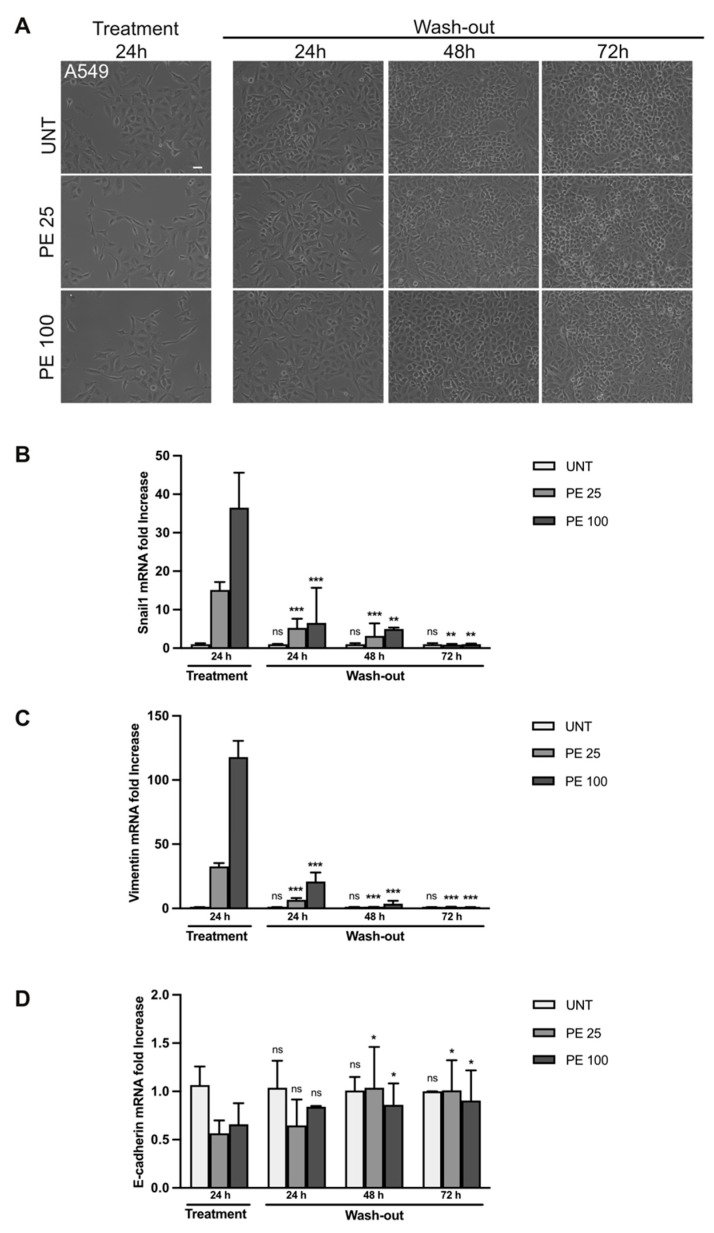
Rescue of PE-induced EMT phenotype in A549 cells. (**A**) Representative phase contrast images of A549 cells untreated (UNT) or treated with PE MNPs (PE 25 and PE 100) for 24 h, and then washed to remove PE-MNPs and cultured in standard media for 24, 48, and 72 h. Bar: 20 μm. (**B**–**D**) Bar graphs (N = 3) showing gene expression levels of the EMT marker Snail1 (**B**), and of the epithelial/mesenchymal markers vimentin (**C**) and E-Cadherin (**D**) in untreated A549 cells or treated with PE MNPsfor 24 h, and after 24, 48, and 72 h of wash-out. Results are expressed as mean value ± SD. ns, not statistically significant; *, *p* < 0.05; **, *p* < 0.01; ***, *p* < 0.001 vs. treatment 24 h.

In order to assess if the cellular and molecular effects of PE MNPs were reversible, we treated BEAS-2B and A549 cell lines for 24 h with PE MNPs and then washed them out by culturing the cells in an MNPs-free medium for 24, 48, and 72 h. Firstly, cell morphology was investigated through phase contrast microscopy. Captured images showed that after PE MNPs removal, both BEAS-2B and A549 cells underwent a phenotypic reversion, losing the spindle-shaped conformation induced by PE MNPs treatment and retrieving their standard organization in compact colonies of polygonal cells (Figure 6A and Figure 7A, respectively). To confirm that these morphological changes were a hallmark of EMT reversion, we assessed via real-time RT-PCR the mRNA expression pattern of key EMT and epithelial/mesenchymal biomarkers, namely Snail1, vimentin, and β4-integrin in BEAS-2B (Figure 6B–D) and Snail1, vimentin, and E-cadherin in A549 cell line (Figure 7B–D). We could observe that, in BEAS-2B cells, Snail1 expression induced by the low dose of PE MNPs significantly decreased already at 24 h after the wash-out, reaching the basal levels of untreated cells after 48 and 72 h, whereas the effects of the high dose (PE 100) were significantly reverted starting from 48 h of wash-out (Figure 6B). Accordingly, the increase in the mesenchymal marker vimentin and the decrease in the epithelial marker β4-integrin induced by PE MNPs treatments were progressively reverted after MNPs removal, reaching the basal levels of untreated cells after 48 h (Figure 6C,D). As for A549 cells, all the selected EMT hallmarks reached their basal expression levels after 72 h of wash-out (Figure 7B–D).

### 2.4. EMT Induction Is Accompanied by an Increase in Migration Capacity but Is Not Associated with Increased Cellular Invasiveness

**Figure 8:**
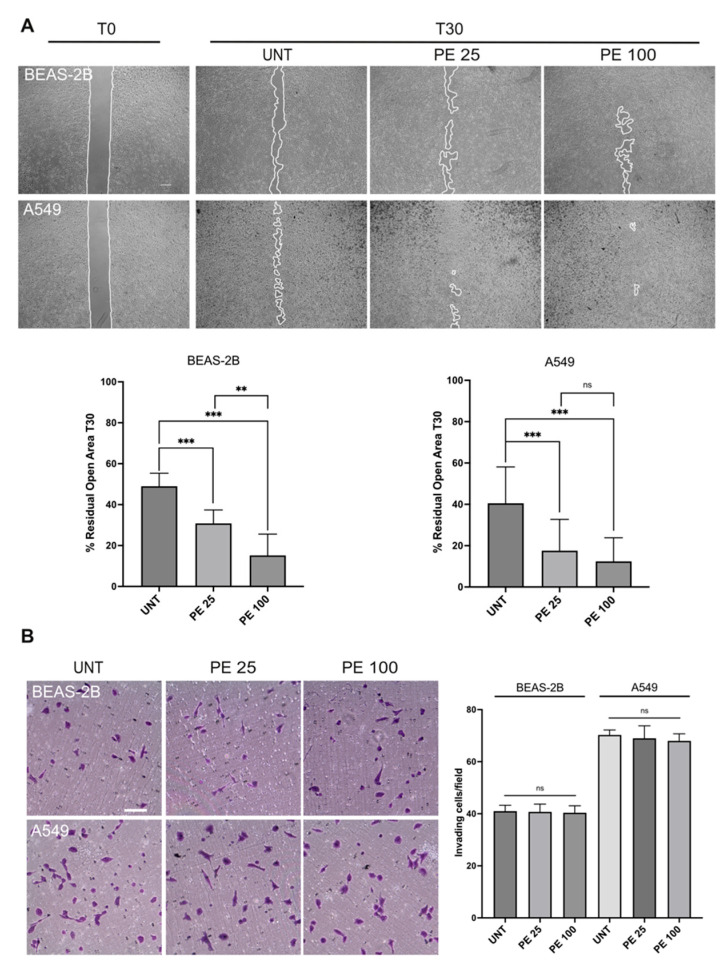
Effects of PE MNPs exposure on BEAS-2B and A549 migration and invasiveness abilities. (**A**) Representative images of scratch assay (N = 3) showing the effect of PE MNPs on BEAS-2B and A549 migration. Bar: 500 μm. The percentage of residual open area after 30 h of treatment with PE MNPs (PE 25 and PE 100), compared to that of untreated cells (UNT), was measured via ImageJ 1.54j software. Results are expressed as mean value ± SD. ns, not statistically significant; **, *p* < 0.01; ***, *p* < 0.001. (**B**) Representative images and quantification of transwell invasion assay (N = 3) on BEAS-2B and A549 treated with PE MNPs (PE 25 and PE 100) for 24 h or untreated (UNT). Migrated cells were stained with crystal violet. Bar: 100 μm. Invasiveness was calculated by counting the number of migrated cells as reported in the Material and Methods section. Results are expressed as mean value ± SD. ns, not statistically significant.

The data analyzed so far have allowed us to observe a strong induction of the EMT process in our cell lines treated with PE MNPs, which can be potentially associated with an increase in the migratory capacity of the cells [16]. Based on this evidence, we aimed to understand whether PE MNPs are also able to trigger an increase in cell motility, using the “scratch assay” as previously described [17]. Interestingly, the treatment with PE MNPs induced increased cell migration when compared to untreated cells. In fact, 30 h after the scratch, the closed area was greater in PE MNPs-treated plates (% residual open area, 31% for 25 μg/mL and 15% for 100 μg/mL vs. 49% of untreated plates) in the BEAS-2B cell line, also showing a dose-dependent response, and the groove was almost completely sealed (17% for 25 μg/mL and 12% for 100 μg/mL vs. 40% of untreated plates) in the A549 treated with both doses (Figure 8A). Thus, PE MNPs treatment, besides conferring to the examined cell lines mesenchymal-like morphological features and altering the expression pattern of well-recognized epithelial and mesenchymal biomarkers reminiscent of EMT, was also able to induce cell migration. Based on this, we further investigated the possible role of PE MNPs treatment in triggering invasive capacity. To investigate this aim, we analyzed the ability of BEAS-2B and A549 cell lines to migrate through transwell Boyden chambers precoated with a thin layer of matrigel that mimics the basement membrane structure in vivo. Upon seeding, cells were serum starved and complete medium was added in the bottom chamber in order to stimulate cell chemotaxis in the presence or absence of PE MNPs treatment. The results showed that all the cell lines exhibited a certain invasive ability, especially the transformed A549 cells, as expected, however, a comparable invasive behavior was observed between cells exposed to PE MNPs and untreated control cells, pointing out the independence of this malignant property from the treatment (Figure 8B).

## 3. Discussion

Inhalation is a major route of human exposure to airborne MNPs [18]. This exposure poses a potential risk for respiratory diseases as inhaled particles can penetrate the respiratory system and reach the alveoli, inducing chronic inflammation, oxidative stress, and cellular damage. These effects may ultimately lead to the development of chronic respiratory conditions [18,19]. Previous studies have demonstrated that MNPs can reach the respiratory epithelium and translocate through several processes, such as diffusion, cellular penetration, or cellular uptake [20]. However, the health risks associated with MNPs inhalation are just beginning to be understood. Reaching a consensus on the impact of MNPs on human health is challenging for the scientific community due to the varying effects MNPs seem to have on different cell types, their differing abilities to be internalized based on size and charge, and the inherent variability of experimental conditions in terms of time of exposure and doses. Most researchers use particles of the most widespread polluting plastic, mainly PS, and of a known size (generally NPs < 1000 nm). The internalization of 50 to 100 nm spherical PS beads has been demonstrated in both BEAS-2B and A549 cells [21]. In vitro studies exploring toxicological effects on BEAS-2B lung epithelial cells have shown that exposure to PS NPs can cause an increase in oxidative stress and inflammatory responses, followed by cell death and epithelial barrier destruction [13]. NPs can also cause mitochondrial dysfunction and damage via overproduction of mitochondrial reactive oxygen species, alteration of the mitochondrial membrane potential, and suppression of mitochondrial respiration [22]. Studies on A549 cells exposed to PS NPs have highlighted reduced cell viability, cell cycle arrest in S phase, higher levels of inflammatory gene transcription, up-regulation of pro-inflammatory cytokines and pro-apoptotic proteins [11], oxidative stress, and lysosomal dysfunction [23,24].

Indeed, it is known that environmental MNPs to which humans are exposed in vivo are highly heterogeneous in terms of type of plastic, size, and shape. In this scenario, we decided to focus on polyethylene (PE) due to the diffusion of this polluting plastic in the environment, and to use a mixture of nano/microparticles (range 200 to 9900 nm). Such PE MNPs have been previously investigated by our group in relation to dermal exposure, by assessing their potential toxicity in vaginal keratinocytes [15]. Regarding the choice of MNPs doses, it is noteworthy that the real concentration of MNPs to which humans are exposed in different conditions cannot be precisely determined. Indeed, the two doses used herein (25 and 100 µg/mL) were higher than environmentally relevant conditions in order to facilitate the observation of any biological effect, in line with other studies reported in the literature [25,26,27]. Moreover, using a low and a high concentration can be representative of different levels of exposure (e.g., general population and plastic industries workers), to understand a possible dose-response action and to fill the gap with respect to previous studies on lung absorption of MNPs that were exclusively focused on industrial workers [28]. To date, only a few studies have explored the impact of PE MNPs on the respiratory epithelium. Due to their origin and characteristics, the human bronchial epithelial cell line BEAS-2B [29] cells and the human alveolar type II cancer cell line A549 [30] are considered valuable tools in mimicking the respiratory epithelium and are currently used to test the effects of MNPs on the respiratory tissue.

In this work, we performed in vitro assays, testing PE MNPs on these widely used cellular models in order to investigate changes in terms of viability and proliferation, cell morphology, migratory ability, and cancer-related features. We found that both low and high doses of PE MNPs did not significantly affect viability and proliferation after short exposure (24 h) nor after longer exposures (48 h and 72 h) in both cell types. This can be considered in line with a previous study testing PE MPs on A549, showing that the higher dose (1000 µg/mL; tested range 1 to 1000 µg/mL) caused only slightly reduced cell viability [31]. Despite this, cell morphology was significantly affected, with treated cells reducing growth in tightly packed clusters with close cell-to-cell contacts and losing their cobblestone shape. Interestingly, MNPs-treated cells showed an enhancement of actin-rich cytoplasmic projections, assuming a more spindle-shaped morphology and more isolated growth behavior, with thick bundles of stress fibers. These changes, visible in both cell lines but particularly evident in A549 cells, as expected, are suggestive of an actin cytoskeleton reorganization compatible with an epithelial-to-mesenchymal transition (EMT) process.

Indeed, it has been shown that MNPs can induce pulmonary fibrosis in mice [32]. Moreover, exposure to PS NPs has been shown to induce apoptosis and inflammatory response in A549 cells, indicating a potential risk of triggering pulmonary fibrosis in humans [11]. Previous studies have demonstrated that the treatment with other MNPs (mainly polystyrene) can improve tumor cell migration, potentially promoting metastasis development and thus contributing to cancer disease progression [33,34]. Moreover, the EMT process, which represents a crucial preliminary step in developing pulmonary fibrosis and cancer, has been documented in A549 cell lines exposed to PS NPs [33].

It is now well known from the literature that the phenotypic changes occurring during EMT are the result of a gene reprogramming that involves numerous transcription factors [16]. Among the most important transcription factors which spatially and temporally cooperate in the EMT induction, Snail1, Snail2, and ZEB1 are well recognized as master transcription factors, mainly related to the early steps of the process [16]. In our study, the occurrence of EMT upon PE MNPs treatment in BEAS-2B and A549 cells was supported by the analysis of such transcription factors and also of specific mesenchymal and epithelial markers, with some differences between cell types that could in part be explainable by the different nature in terms of tumor transformation of these cell lines. Moreover, the rescue assay performed by washing out PE MNPs after 24 h treatment further confirmed that the induction of EMT transition was mediated by acute PE MNPs exposure, and also pointed out the ability of respiratory epithelium to recover from MNPs-induced response within 72 h of the MNPs removal, at least at the tested doses and exposure times. To our knowledge, this is the first time the EMT process caused by PE MNPs exposure has been investigated on both tumoral and untransformed respiratory epithelial cells.

Furthermore, MNPs exposure has been linked to the onset of different types of cancer, including lung, skin, and digestive system cancer, in line with the most common entry routes for these particles. Among the potential mechanisms involved in MNPs-induced carcinogenesis are cytotoxicity, which is able to lead the activation of programmed cell death pathways, in addition to the induction of DNA damage with subsequent accumulation of mutations, as well as the release of reactive oxygen species (ROS) triggering oxidative stress and tissue inflammation [28]. Previous data also suggest that MNPs can induce epigenetic alterations and can alter microRNA expression [15,35,36] which might potentially contribute to cancer initiation and progression. Indeed, the role of EMT in the tumorigenesis of different cancers, including lung [37], and the association of E-cadherin loss and vimentin expression with the malignant phenotype of non–small cell lung cancer (NSCLC) [38,39] is well known.

In this view, our results indicate that EMT induction by PE MNPs can represent a notable key to understanding the correlation between MNPs and lung cancer. Considering the pivotal role of EMT in the first steps of the metastatic cascade, represented by the acquisition by tumor cells of the ability to migrate and invade the extracellular matrix [40], we investigated if PE MNPs exposure could change the migratory capacity of respiratory epithelial cells. Therefore, we found a significantly enhanced migration in both BEAS-2B and A549 cells upon PE MNPs treatment. However, further analyses of the invasive potential via transwell assays underlined no significant improvement of respiratory cells invasiveness. Such data suggest that the cellular response to PE MNPs exposure described in this study (morphological changes, actin cytoskeleton reorganization, EMT induction, and increased migration) does not necessarily result in an escalation of tumorigenic features involved in the metastatic process.

In conclusion, the present study represents a significant step towards the elucidation of the real impact of MNPs exposure on human health, particularly in relation to the inhalation route of entry and in the context of carcinogenesis. The data obtained using a type of plastic (PE) that is still little investigated and a combination of nano/microplastic composition used at low/high doses of exposure that better mimics environmental conditions, might also contribute to overcoming some limitations of previous research on this topic. Of course, future analyses should be performed to further disclose the molecular mechanisms at the basis of respiratory epithelium response to MNPs and the potential implications for lung health.

## 4. Materials and Methods

### 4.1. Preparation of Polyethylene Micro/Nanoplastics (PE MNPs)

Polyethylene micro/nanospheres (PE MNPs) of 200 to 9900 nm (Cospheric LLC, Santa Barbara, CA, USA) solutions were prepared as previously described [15]. Briefly, dry powder was resuspended in ultrapure nuclease-free ddH2O with 1% Tween20 (Dako, Agilent Technologies, Santa Clara, CA, USA) to obtain a 100 mg/mL stock solution. For cell treatments, a working solution with a 10 mg/mL concentration of PE MNPs was produced by diluting the stock with nuclease-free ddH_2_O. Both stock and working solution were stored at 4 °C until use.

### 4.2. Cell Culture and PE MNPs Treatment

The human bronchial epithelial cell line BEAS-2B (CRL-3588™) and the human lung carcinoma cell line A549 (CCL-185™) were purchased from ATCC (Manassas, VA, USA). For the BEAS-2B culture, RPMI-1640 Medium (Sigma-Aldrich, Merck KGaA, Darmstadt, Germany) was used, supplemented with 10% fetal bovine serum (Gibco, Thermo Fisher Scientific, Waltham, MA, USA), 1 mm sodium pyruvate (Gibco, Thermo Fisher Scientific, Waltham, MA, USA), 100 U/mL penicillin, 100 mg/mL streptomycin, and 2 mM L-glutamine (Gibco, Thermo Fisher Scientific, Waltham, MA, USA). For the A549 cells culture, DMEM high glucose (Sigma-Aldrich, Merck KGaA, Darmstadt, Germany) was used, supplemented with 10% fetal bovine serum, 100 U/mL penicillin, 100 mg/mL streptomycin, and 2 mM L-glutamine (Gibco, Thermo Fisher Scientific, Waltham, MA, USA). Cell lines were maintained at 37 °C in an incubator with a 5% CO_2_-humidified atmosphere. For cell treatments, the working solution of PE MNPs was added to the respective cell culture medium, after vortexing and pipetting, to obtain a PE MNPs final concentration of 25 µg/mL (PE 25) and 100 µg/mL (PE 100). For the untreated control (UNT), cell culture media were added with ultrapure 1% Tween20 (Dako, Agilent Technologies, Santa Clara, CA, USA) solution to a final concentration of 0.01%, achieving the same surfactant concentration as treated cells.

### 4.3. Cytotoxicity Assay (MTT)

To assess viability and uncover possible cytotoxic effects, BEAS-2B and A549 cells were seeded in 96-well plates with 4 × 10^3^ cells/well in 150 µL of medium, and incubated for 24 h at 37 °C. Cells were then treated with PE MNPs (25 µg/mL and 100 µg/mL) for 24, 48, or 72 h. After treatment, a 5 mg/mL MTT solution (Sigma-Aldrich, Merck KGaA, Darmstadt, Germany) was added to achieve a final concentration of 0.5 mg/mL, and the plates were then incubated for 3 h at 37 °C. The medium was then removed and 100 μL of dimethyl sulfoxide (DMSO) (SERVA Electrophoresis GmbH, Heidelberg, Germany) per well was added and incubated for 5 min at 37 °C. The absorbance was then measured at OD = 550 nm using a microplate reader (Bio-Rad, Hercules, CA, USA). Cell viability in control samples (DMSO) and PE MNPs-treated cells was reported as OD values, having six determinations per assay for each experimental condition in three independent experiments.

### 4.4. Proliferation Assay

To measure cellular proliferation, BEAS-2B and A549 cells were seeded in 24-well plates with 25 × 10^3^ cells/well in 1.5 mL of medium, and incubated for 24 h at 37 °C. Cells were then treated with PE MNPs solutions (25 µg/mL and 100 µg/mL) for 24, 48, or 72 h. After treatment, Trypan blue was added to the cells in a 1:1 ratio, and counts were performed manually using a Neubauer-improved chamber and an optical microscope. Viable cell numbers were assessed in two independent experiments, with three determinations for each experimental condition in each experiment.

### 4.5. Fluorescence Microscopy and Imaging Quantification

BEAS-2B and A549 cells were seeded on 2% gelatin-coated glass coverslips (12 mm, Cover slips; Epredia, Germany) in 24-well plates (5 × 10^4^ cells/well) and grown for 24 h. Cells were then treated with PE MNPs solutions (25 µg/mL and 100 µg/mL) for 24 h. After treatment, cells were fixed in 4% paraformaldehyde. Immunofluorescence and imaging were performed as previously described [41]. Actin filaments and nuclei were stained via incubation with TRITC-Phalloidin (Cat. No. P1951; 1:100 dilution; Sigma-Aldrich, Merck KGaA, Darmstadt, Germany) and 4′, 6-diamidino-2-phenylindole dihydrochloride (DAPI) (Sigma-Aldrich, Merck KGaA, Darmstadt, Germany), respectively. Images were captured with an ApoTome.2 (Zeiss, Oberkochen, Germany) fluorescence imaging system, using a 20× lens, and processed with Zen 3.10 microscopy software (Zeiss). Quantitative analysis of the fluorescence intensity was performed via Zen 3.10 microscopy software (Zeiss), analyzing 5 different fields per condition, randomly taken from 3 independent experiments.

### 4.6. Quantitative Real-Time PCR (qRT-PCR)

For qRT-PCR analysis, BEAS-2B and A549 cells were seeded (3 × 10^5^ cells/well) in 6-well plates. After 24 h of incubation at 37 °C, cells were treated with PE MNPs solutions (25 µg/mL and 100 µg/mL) for 24 h. Media was then removed and total RNA was extracted with Quick-RNA™ Microprep Kit (Zymo Research, Irvine, CA, USA) by applying standard protocol. RNA concentration and purity was measured using the Nanodrop 1000 (Thermo Fisher Scientific, Waltham, MA, USA). RNA integrity was assessed by running samples on a 0.8% agarose gel. One microgram of total RNA was reverse transcribed using the iScriptTM cDNA Synthesis Kit (Bio-Rad), with a mixture of oligo(dT) and random primers. qRT-PCR was performed in triplicate with SYBR Green (Bio-Rad) for the genes of interest CDH1, CDH2, SNAI1, SNAI2, ITGB4, VIM, ZEB1, and the housekeeping gene RNA18SN1 (Table S1), using 15 ng of cDNA, on a QuantStudio 1 real-time PCR system (Thermo Fisher Scientific, Waltham, MA, USA). The results of three independent experiments were analyzed using the 2^−ΔΔCt^ method as reported [42].

### 4.7. Rescue Assay for PE-Induced Cellular and Molecular EMT Phenotype

BEAS-2B and A549 cells were seeded (1 × 10^5^ cells/well) in 6-well plates and incubated at 37 °C. After 24 h, cells were treated with PE MNPs solutions (25 µg/mL and 100 µg/mL) for 24 h. Then, a wash-out procedure was performed as described. PE-added media were removed, cells were washed twice in PBS, and standard culture media were added for 24, 48, and 72 h of growth. After RNA extraction, qRT-PCR for the genes of interest CDH1, CDH2, SNAI1, ITGB4, and VIM was carried out as described above.

After treatment and at every time point of PE-wash-out, cell morphology was analyzed by capturing images with EVOS xl transmitted light microscope (AMG, Thermo Fisher Scientific, Waltham, MA, USA) with a 20× objective.

### 4.8. Protein Isolation and Western Blot Analysis

For Western blot experiments, BEAS-2B and A549 cells were seeded 1 × 10^6^ cells in 100 mm cell culture dishes. Cells were lysed and whole protein content was extracted using ELB Buffer (250 mm NaCl, 50 mm Hepes pH7.5, 5 mm EDTA, 1% NP-40), completed with phosphatase and protease inhibitors. A total of 30 to 40 μg of protein was separated on a 6% or 12% SDS-PAGE gel and Western blot analysis was performed as previously described [43]. Analysis of expression level of target proteins E-cadherin, N-cadherin, β4-integrin, vimentin, and internal control heat shock protein HSP90 was performed using the ImageJ 1.54j software. Antibodies and relative concentrations are listed in Table S2.

### 4.9. Wound Healing Assay

To assess the migratory capacity of BEAS-2B and A549, cells were seeded in a 2-well insert (Ibidi GmbH, Gräfelfing, Germany) placed in a 12-well plate, with a density of 3.5 × 10^4^ cells in 70 µL of medium per well, and grown at 37 °C for 24 h. Insert was then removed and cells were treated with PE MNPs solutions (25 µg/mL and 100 µg/mL) until the wound healing process was completed (30 h). Images at T0 and T30 of three independent experiments, each with two wound fields/condition were captured with an EVOS xl transmitted light microscope (AMG, Thermo Fisher Scientific, Waltham, MA, USA) with a 4× objective. ImageJ 1.54j software was used to measure the surface area of the scratch (with reference to the mark, which had been drawn on the well) and these measurements were used to calculate the percentage of the residual open area at T30.

### 4.10. Transwell Invasion Assay

To evaluate the effects of PE MNPs on cell invasiveness, a transwell Boyden insert membrane (8 µm pore size; Falcon, Corning, AZ, USA) was precoated with 20 μL of Matrigel solution (BD Biosciences, San Jose, CA, USA), placed in a 24-well plate and incubated for 5 min at RT, for gelling. Medium containing 10% FBS was added in the lower chamber. BEAS-2B and A549 cells were seeded into the upper chamber at a density of 2.5 × 10^4^ cells/chamber, and maintained in 200 μL of serum-free medium. Cells were treated with PE MNPs solutions (25 µg/mL and 100 µg/mL) for 24 h, then fixed with 4% paraformaldehyde (PFA) and stained with 0.1% crystal violet solution (BD Biosciences, San Jose, CA, USA) for 30 min. Different fields of cells were randomly selected from three independent experiments and photographed under a light microscope. Cells from three microscope fields per condition were counted and the results were plotted in a graph.

### 4.11. Statistical Analysis

Data are presented as the mean ± standard deviation (SD) of three independent experiments and were performed in triplicate, unless otherwise specified. Statistical analyses were performed using GraphPad Prism 8.0.1 (GraphPad Software Inc., San Diego, CA, USA). For qRT-PCR and immunofluorescence, quantification signal data were analyzed using the Brown–Forsythe and Welch ANOVA test. For Western blot, MTT, invasion test, and scratch test, data were analyzed using ordinary one-way analysis of variance (ANOVA) followed by the Tukey’s multiple comparison test. A P-value less than 0.05 was considered statistically significant.

## Data Availability

All the data are provided in the article.

## Supplementary Materials

The supporting information can be downloaded at: https://www.mdpi.com/article/10.3390/ijms251810168/s1.
