# Supplementary material for: Polyethylene Micro/Nanoplastics Exposure Induces Epithelial–Mesenchymal Transition in Human Bronchial and Alveolar Epithelial Cells
Source: Int J Mol Sci. 2024 Sep 22;25(18):10168. doi: 10.3390/ijms251810168 (PMC11432389; doi:10.3390/ijms251810168)
Supplement: Supplementary file 1 [file ijms-25-10168-s001.zip › ijms-3169065-supplementary.pdf]

Supplementary Material

Table S1: Quantitative real-time PCR primer sequences and annealing temperature

Gene symbol	Forward primer (5'-3')	Reverse primer (5'-3')	Amplicon length (bp)	Annealing temperature (°C)
CDH1	TGGAGGAATTCTTGCTTTGC	CGCTCTCCTCCGAAGAAAC	64	60
CDH2	CTCCATGTGCCGGATAGC	CGATTTCACCAGAAGCCTCTAC	92	60
SNAI1	CCTCGTCAGGAAGCCCTC	CTGCTGGAAGGTAAACTCTGGA	85	60
SNAI2	ACACACATACAGTGATTATTTCCCC	AGTGATGGGGCTGTATGCTC	104	60
VIM	AAATGGCTCGTCACCTTCGT	AGAAATCCTGCTCTCCTCGC	113	60
ITGB4	GGGAAAAAGCAAGACCACACC	CCCTCTGTTCCACCTGCTTC	103	60
ZEB1	TGCCAACAGACCAGACAGTG	TTGCCCTTCCTTTCCTGTGTC	95	60
RNA18SN1	AACCAACCCGGTCAGCCCCT	TTCGAATGGGTCGTCGCCGC	119	60

Table S2: Antibodies for Western blot analysis

Protein name	Primary antibody	Ab I dilution	Secondary antibody	Ab II dilution
E-cadherin	Santa Cruz Biotechnology (sc-21791)	1: 200	Jackson ImmunoResearch: horseradish peroxidase (HRP- conjugated anti-mouse)	1: 40000
N-cadherin	Santa Cruz Biotechnology (sc-59987)	1:500	Jackson ImmunoResearch: horseradish peroxidase (HRP- conjugated anti-mouse)	1: 40000
vimentin	Dako (M0725)	1:1000	Jackson ImmunoResearch: horseradish peroxidase (HRP- conjugated anti-mouse)	1: 40000
ß4-integrin	Santa Cruz Biotechnology (sc135950)	1:800	Jackson ImmunoResearch: horseradish peroxidase (HRP- conjugated anti-mouse)	1: 40000
HSP90	Proteintech (13171-1-AP)	1:5000	Advansta: horseradish peroxidase (HRP-conjugated anti-rabbit)	1:10000

.
